# Supplementary material for: A trimeric Rab7 GEF controls NPC1-dependent lysosomal cholesterol export
Source: Nat Commun. 2020 Nov 3;11:5559. doi: 10.1038/s41467-020-19032-0 (PMC7642327; doi:10.1038/s41467-020-19032-0)
Supplement: Supplementary file 1 — Supplementary Information [file 41467_2020_19032_MOESM1_ESM.pdf]

# Supplementary Information

**A trimeric Rab7 GEF controls NPC1-dependent lysosomal cholesterol export**
*van den Boomen et al. Nat. Commun. 2020*

Contains:
- Supplementary Figures 1-10
- Supplementary Methods

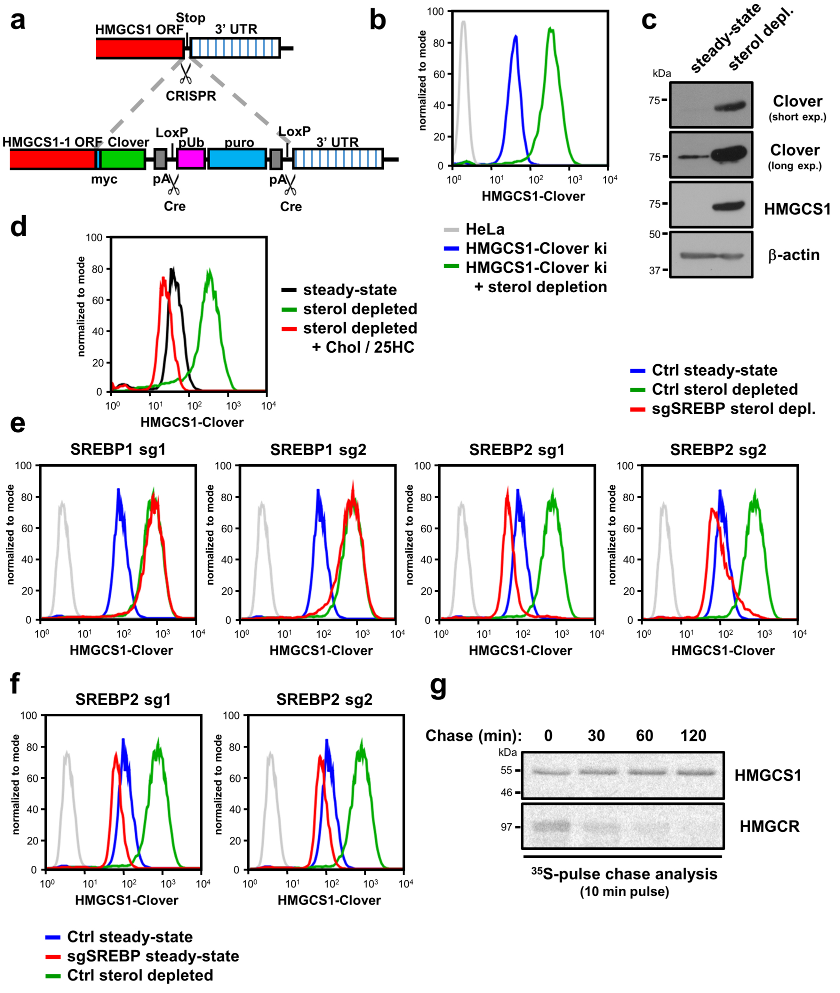


**Supplementary Fig. 1: Characterisation of the HMGCS1-Clover cholesterol reporter.**
**a** Schematic for the generation of an HMGCS1-Clover knock-in in HeLa cells. **b-c** Sterol depletion induces HMGCS1-Clover expression. HeLa HMGCS1-Clover cells were sterol depleted overnight (green) or left untreated (blue) and HMGCS1-Clover expression was analysed by flow cytometry (3 independent experimental replicates) (**b**) or immune blotting using GFP (Clover) and HMGCS1-specfic antibodies (**c**). **d** Sterol addition reverses depletion-induced HMGCS1-Clover upregulation. HMGCS1-Clover cells were sterol depleted in the presence (red) or absence (green) of cholesterol and 25-hydroxy-cholesterol and HMGCS1-Clover was monitored using flow cytometry **e-f** Sterol-depletion induced HMGCS1 up-regulation depends on SREBP2. HMGCS1-Clover CAS9 cells were transfected with two independent sgRNAs against *SREBF1* or *SREBF2* (red lines) or control (blue and green lines), sterol depleted overnight where indicated and analysed by flow cytometry for HMGCS1-Clover expression at day 8. **g** HMGCS1 protein stability is not cholesterol-sensitive. HeLa cells were sterol depleted overnight, pulse-labelled with $^{35}$S-Met/Cys and chased in the presence of excess cholesterol/25-hydroxy-cholesterol for the indicated times. After cell lysis, HMGCS1 or HMGCR was immune precipitated, separated by SDS-PAGE and analysed by autoradiography.

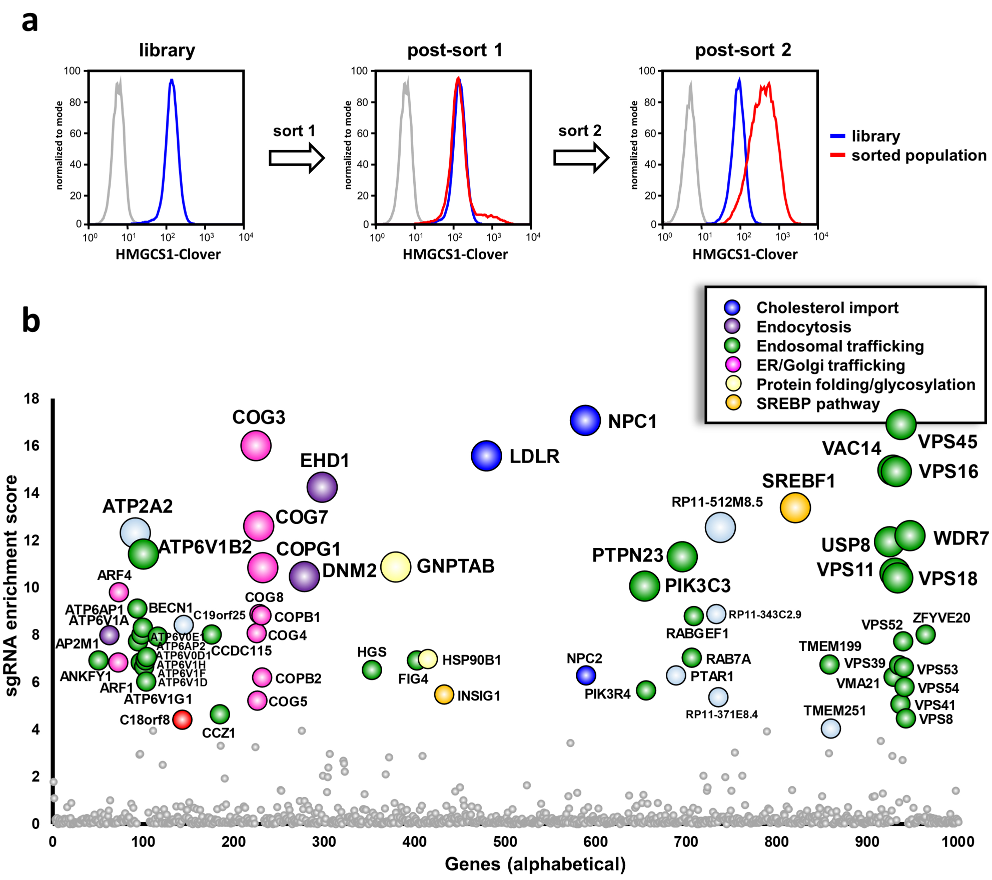


**Supplementary Fig. 2: A sub-genomic CRISPR screen for regulators of cellular cholesterol homeostasis.**
**a** HeLa HMGCS1-Clover Cas9 cells were transduced with the top 1000 sgRNA library (12,000 sgRNAs). Rare HMGCS1- Clover$^{high}$ cells were sorted using two rounds of flow cytometry, resulting in a 75% enriched HMGCS1-Clover$^{high}$ population (right panel, red line). **b** Illumina sequencing of sgRNAs in the isolated HMGCS1-Clover$^{high}$ population shows sgRNA enrichment for genes involved in cholesterol uptake (blue), protein folding and glycosylation (yellow), membrane trafficking of LDLR/LDL (pink, purple, green) and SREBP2 function (orange). Genes with MAGeCK sgRNA enrichment score $<10^{-4}$ are annotated by gene name. The full dataset is available in Supplementary Data 1.

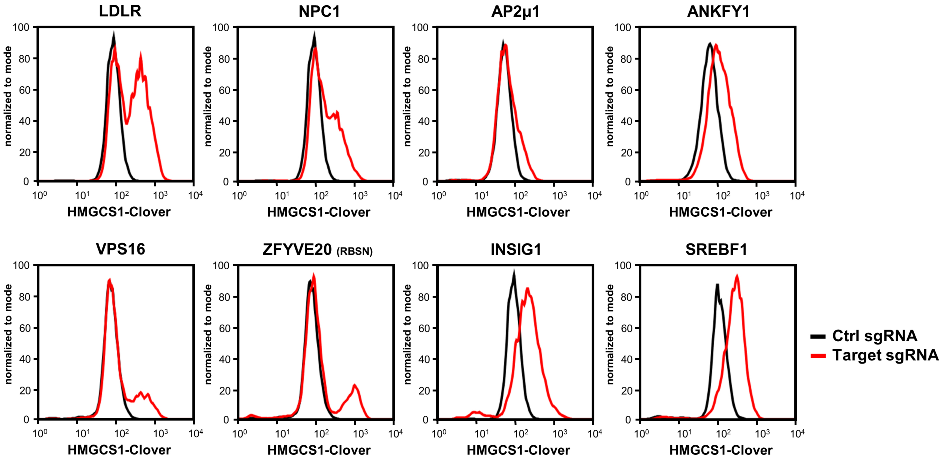


**Supplementary Fig. 3: Validation of select genetic screening hits using the HMGCS1-Clover reporter.**
CRISPR knockout of select screening hits induces spontaneous HMGCS1-Clover up-regulation. HMGCS1-Clover CAS9 cells were transfected with sgRNAs against indicated targets (red lines) or control (black lines) and HMGCS1-Clover expression was analysed by flow cytometry at day 5 (*AP2μ1*), day 7 (*LDLR, NPC1, INSIG1*), day 8 (*ANKFY1*, *SREBF2*) or day 9 (*ZFYVE20, VPS16*).

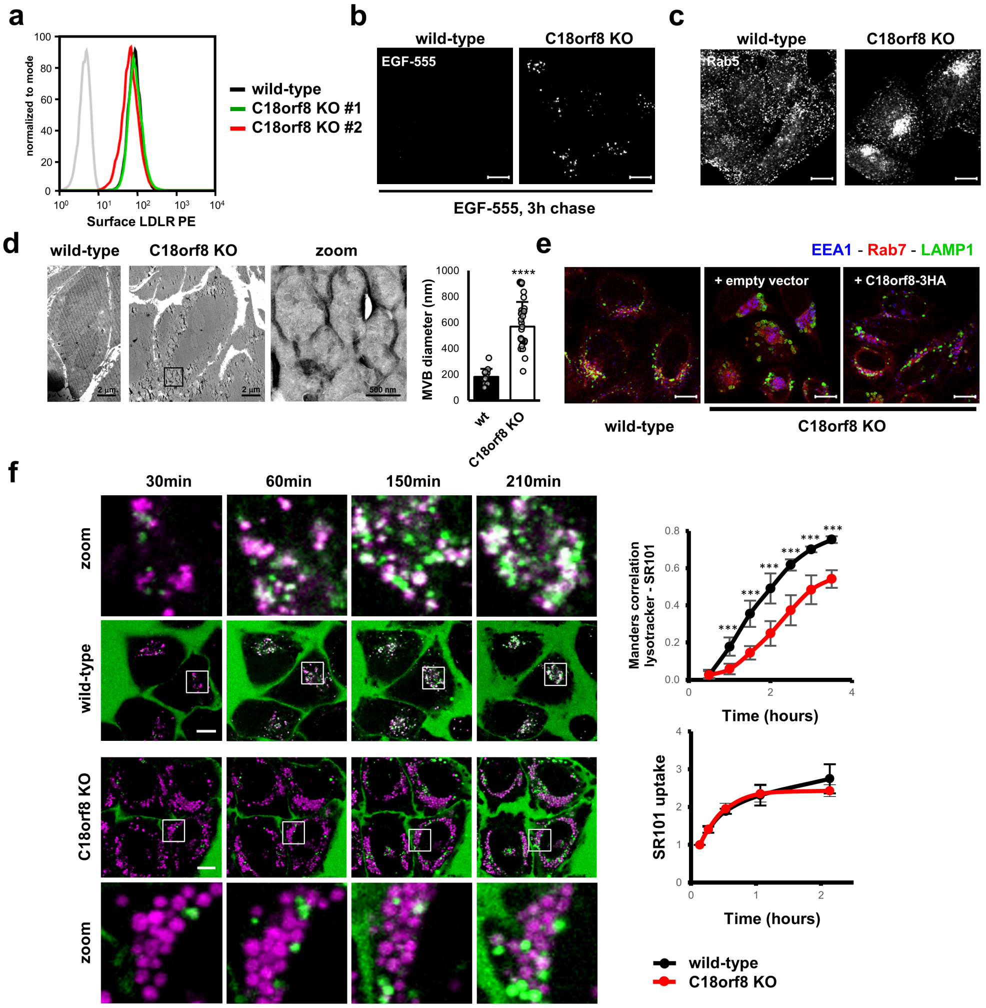


**Supplementary Fig. 4: *C18orf8*-deficient cells show severe defects in late endosome morphology and trafficking.**
**a** *C18orf8*-deficient cells have normal cell surface LDL receptor (LDLR) expression. Wild-type (black line) or *C18orf8*-deficient cells (red and green lines) were labelled for cell surface LDLR and analysed by flow cytometry. **b** *C18orf8*-deficient cells are defective in EGF degradation. Wild-type or *C18orf8*-deficient cells were pulse-labelled with AlexaFluor-555 conjugated EGF, incubated for 3 hours and fixed. Remaining EGF-555 fluorescence was visualised by confocal microscopy. **c** *C18orf8*-deficient cells show clustering of early endosomes (EE). Rab5 staining of wild-type and *C18orf8*-deficient cells. Representative images are shown from 5 fields per condition. **d** *C18orf8*-deficient cells accumulated enlarged multivesicular bodies (MVBs). Electron microscopy of wild-type and *C18orf8*-deficient cells. MVB size was quantified in n=30 cells. Error bars reflect standard deviation (two-sided unpaired t-test, **** p<0.0001). **e** Complementation of endosomal phenotypes in *C18orf8*-deficient cells. Wild-type cells, *C18orf8*-deficient cells and *C18orf8*-deficient cells complemented with C18orf8-3xHA were labelled for EEA1 (blue), Rab7 (red) and LAMP1 (green) and visualised by confocal microscopy. Representative images are shown from 5 fields per condition. **f** *C18orf8*-deficient cells show delayed SR101 trafficking into the late endosomal compartment. Wild-type or *C18orf8*-deficient cells were labelled with lysotracker (magenta), incubated with Sulforhodamine 101 dye (SR101, green) and imaged live for 3.5h at 1.5 min intervals. Select frame overlays from one of 5 positions imaged per experiment per condition are shown. Quantification of SR101 trafficking into the lysotracker+ LE compartment over time is reported as Mander's correlation (top right graph) determined using n=5 fields (containing 10-30 cells per field of view) from 2 independent experiments. Error bars reflect standard deviation (unpaired two-sided t-test, *** p<0.001). SR101 uptake was determined at indicated times by flow cytometry (bottom right graph). Scale bars for confocal microscopy = 10µm, scale bars for EM = 2µm / 500nm as indicated.

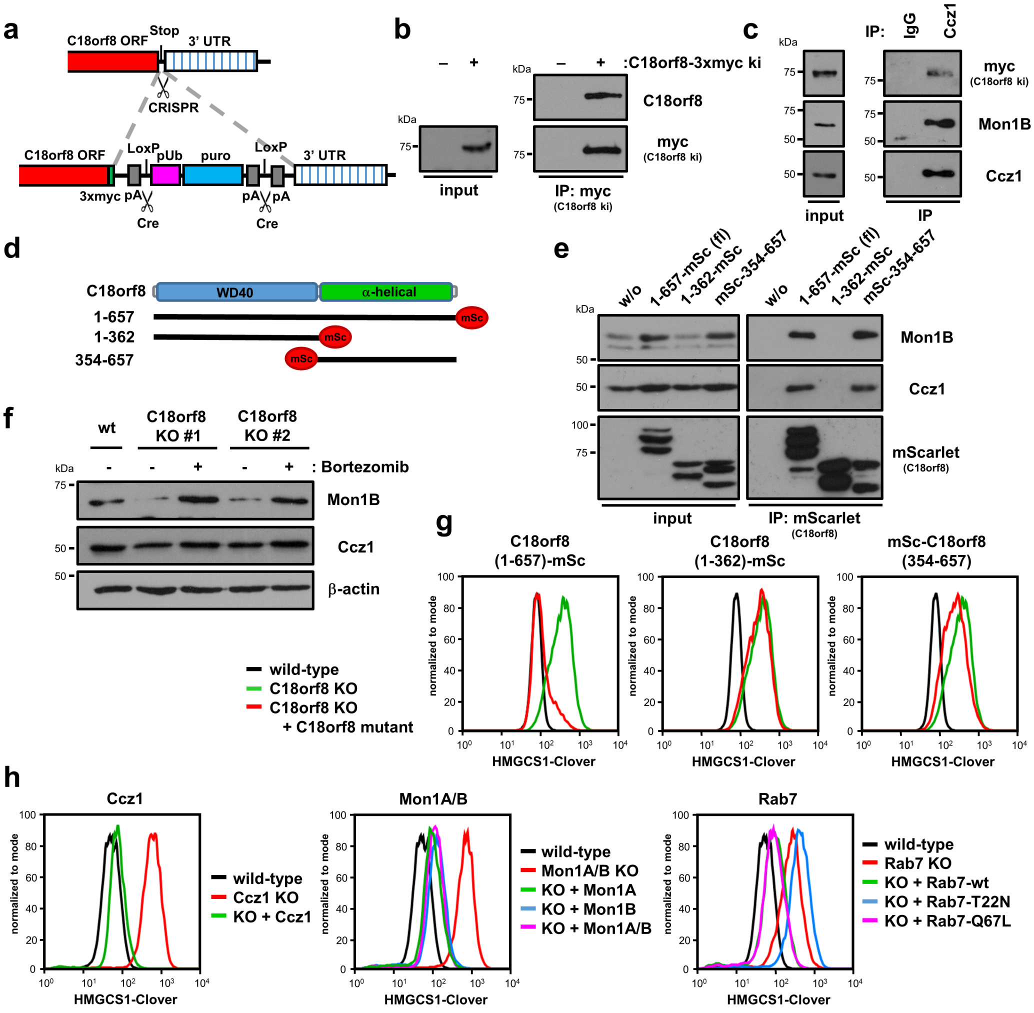


**Supplementary Fig. 5: C18orf8 interacts with the mammalian Mon1-Ccz1 (MC1) complex and is essential for complex stability.** **a-c** Endogenous C18orf8 interacts with the mammalian MC1 complex. **a** Schematic for generation of an endogenous C18orf8-3xMyc fusion protein in HeLa cells. **b** Myc immune precipitation and immunoblotting of a knock-in clone shows the endogenous C18orf8-3xMyc fusion protein, detected by both C18orf8 and Myc antibodies. **c** Immune precipitation using a Ccz1-specific antibody but not an isotype control shows an interaction between Ccz1, endogenous C18orf8-3xMyc and Mon1B in C18orf8-3xMyc ki cells. **d-e** The C18orf8 C-terminal α-helical domain interacts with and stabilizes mammalian MC1. **d** Depiction of C18orf8 domain structure and mutants created. **e** *C18orf8*-deficient cells were complemented with mScarlet-labelled C18orf8 full-length (wt), N-terminal (AA 1-362) and C-terminal domains (AA 354-657). mScarlet proteins were immune precipitated and immune precipitations and input controls were analysed by immune blotting using Mon1B, Ccz1 and mScarlet/RFP-specific antibodies. Note wild-type C18orf8 and its C-terminal domain (AA 354-657) stabilize Mon1B expression in input controls. **f** Mon1B expression in *C18orf8*-deficient cells is rescued by overnight proteasome inhibition (bortezomib). **g** The C18orf8 C-terminus alone is insufficient to complement *C18orf8* functions. *C18orf8*-deficient cells (green) were complemented with mScarlet-tagged C18orf8 full-length (wt), N-terminal (AA 1-362) and C-terminal domains (AA 354-657) (red lines) and analysed by flow cytometry for HMGCS1-Clover expression. **h** Complementation of *Ccz1*-, *Mon1A/B*- and *Rab7*-deficient cells. *Ccz1*-, *Mon1A/B*- and *Rab7*-deficient cells (red lines) were transduced respectively with Ccz1-3xMyc (left panel, green line); Mon1A-3xMyc (middle panel, green line), Mon1B-3xMyc (blue line) or both (magenta line); and 2xHA-Rab7-wt (right panel, green line), -T22N (blue line) or -Q67L (magenta line) and HMGCS1-Clover expression was analysed using wild-type HMGCS1-Clover cells (black line) as a control.

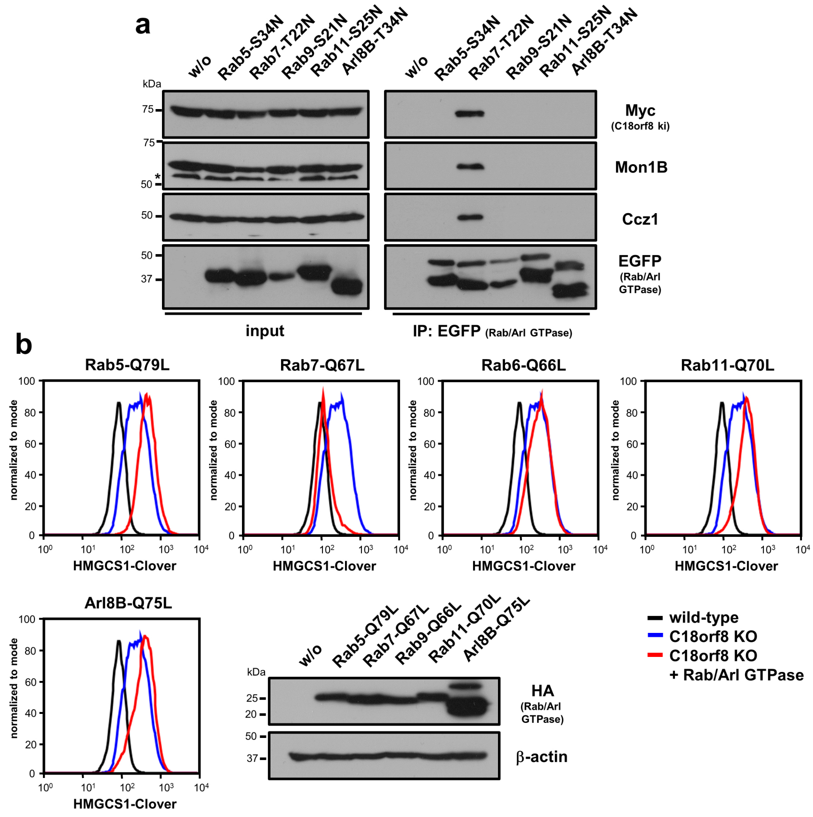


**Supplementary Fig. 6: The Mon1-Ccz1-C18orf8 (MCC) complex specifically regulates Rab7**.

**a** The MCC complex specifically interacts with Rab7. C18orf8-3xMyc knock-in cells were transduced with EGFP-tagged dominant-negative Rab5-S34N, Rab7-T22N, Rab9-S21N, Rab11-S25N or Arl8B-T34N. EGFP immune precipitation were analysed by Western blotting using Myc-, Mon1B-, Ccz1- and GFP-specific antibodies (2 independent experimental replicates). **b** Cholesterol defects in *C18orf8*-deficient cells are restored by overexpression of a constitutively active Rab7 (Q67L), but not other endosomal GTPases. *C18orf8*-deficient cells (blue lines) were transduced with HA-tagged constitutively active Rab5-Q75L, Rab7-Q67L, Rab9-Q66L, Rab11-Q70L and Arl8B-Q75L (red lines) and analysed at day 6 by flow cytometry for HMGCS1-Clover expression (2 independent experimental replicates). Expression of the HA-tagged GTPases was confirmed by Western blotting.

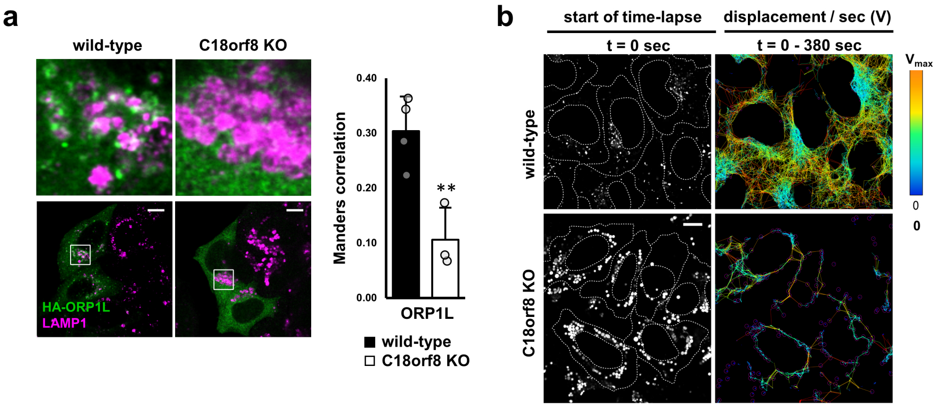


**Supplementary Fig. 7: *C18orf8*-deficient cells show impaired ORP1L recruitment and reduced late endosome motility.**
**a** Wild-type and *C18orf8*-deficient cells were transfected with HA-ORP1L and stained intracellularly for HA (green) and LAMP1 (magenta). Mander's correlation was determined for n=4 fields from 2 independent experiments. Error bars reflect standard deviation (two-sided unpaired t-test, $p=8.3\times10^{-3}$ ** $p<0.01$). **b** Wild-type or *C18orf8*-deficient cells were labelled with lysotracker and lysotracker+ endosomes (white) were traced for 380 sec using live cell microscopy at 3 sec intervals. Displacement of individual endosomes was calculated using TrackMate for Fiji and is depicted with maximum velocity of travel indicated by a heat map (blue: no motility, red: maximum motility attainable under selected tracking parameters).

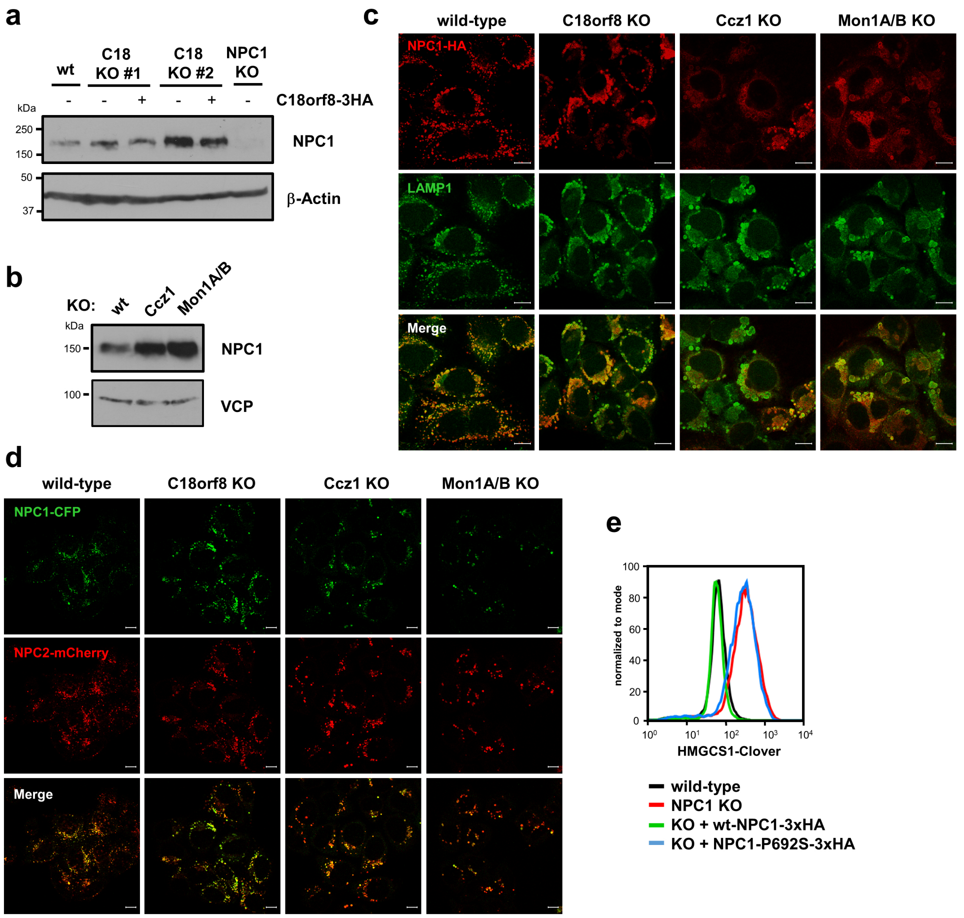


**Supplementary Fig. 8: NPC1 expression and localisation is unperturbed in *C18orf8*-, *Ccz1*- and *Mon1A/B*-deficient cells.**
**a-b** *MCC*-deficient cells show normal to increased NPC1 expression. Immune blotting for endogenous NPC1 in (**a**) wild-type, *C18orf8*-deficient and complemented *C18orf8*-deficient cell clones; or (**b**) wild-type, *Ccz1*- and *Mon1A/B*-double deficient cells. **c-d** NPC1 localisation is largely unperturbed in *MCC*-deficient cells. **c** NPC1 localises to LAMP1+ LE/Ly in *MCC*-deficient cells. Wild-type, *C18orf8*-, *Ccz1*- and *Mon1A/B*-deficient cells were transduced with the inactive NPC1-P692S-3xHA and stained for HA (red) and LAMP1 (green). **d** NPC1 co-localises with the NPC2 cholesterol carrier in *MCC*-deficient cells. Wild-type, *C18orf8*-, *Ccz1*- and *Mon1A/B*-deficient cells were stably transduced with NPC1-P692S-CFP (green) and NPC2-mCherry (red) and monitored using confocal microscopy. Representative images are shown of 5 fields per condition. Scale bars = 10μm. **e** Complementation of *NPC1*-deficient cells. *NPC1*-deficient cells (red line) were transduced with wild-type (green line) or P692S mutant NPC1-3xHA (blue line) and analysed by flow cytometry for HMGCS1-Clover expression.

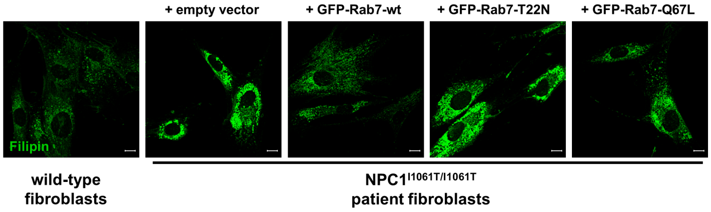


**Supplementary Fig. 9: Rab7 overexpression reduces cholesterol accumulation in Niemann Pick primary patient fibroblasts.** NPC1$^{I1061T/I1061T}$ primary patient fibroblasts were stably transduced with GFP-Rab7 wild-type (wt), dominant negative (T22N) or constitutively active (Q67L) or empty vector and labelled for free cholesterol at day 7 using Filipin staining. Fibroblast from a healthy individual were used as a control. Overexpression of wild-type Rab7 reduces cholesterol accumulation in NPC patient fibroblasts, whereas Rab7-T22N does not and Rab7-Q67L gives an intermediate phenotype. Representative images are shown from 5 fields per condition and 2 independent experiments. Scale bars = 10μm.

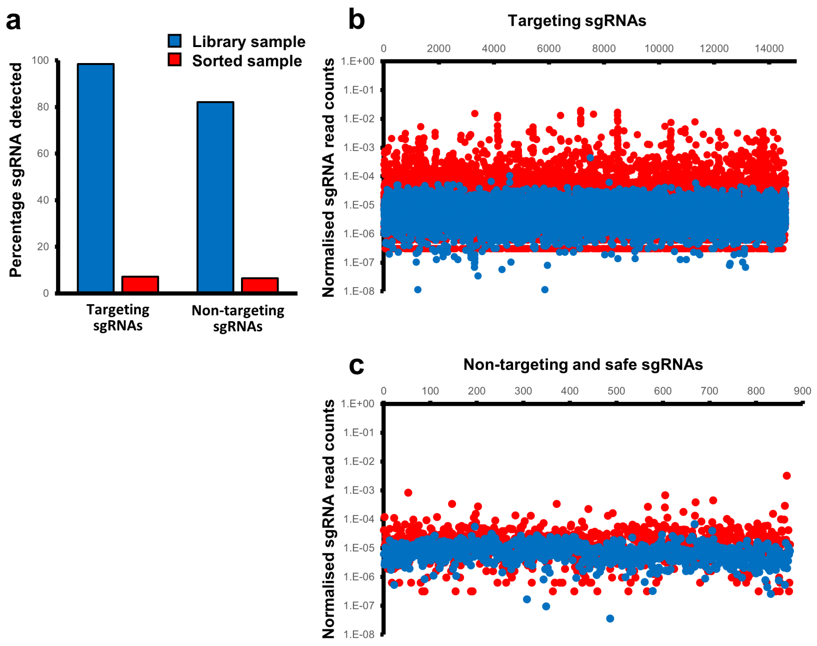


**Supplementary Fig. 10: sgRNA enrichment and de-enrichment during genome-wide CRISPR screening.**
**a** De-enrichment of targeting and non-targeting sgRNAs during phenotypic selection. The percentage of total sgRNAs detected within the library (blue) and enriched sorted sample (red) is indicated. **b** Phenotypic selection strongly enriches the normalized read counts for a substantial number of targeting (top panel) but not non-targeting sgRNAs (bottom panel). This enrichment yields genetic screening hits. Read counts for individual targeting and non-targeting sgRNAs are shown as a percentage of total read counts within the library (blue) or sorted sample (red). To allow comparison between the library and sorted sample, normalized read counts are only shown for sgRNAs that are present in both samples (14,587 targeting, 873 non-targeting).

# Supplementary methods

## Materials

### Plasmid DNA

| Construct | Description | Origin |
|---|---|---|
| pHRSIN.pSFFV MCS pGK Puro | lentiviral parent vector, puromycin resistance | [1] |
| pHRSIN.pCMV EGFP pGK Puro | lentiviral parent vector, puromycin resistance, CMV promoter | [1] |
| pHRSIN.pSFFV MCS pGK Hygro | lentiviral parent vector, hygromycin resistance | [1] |
| pHRSIN.pSFFV MCS pSV40 Blast | lentiviral parent vector, blasticidin resistance | [1] |
| pHRSIN.pSFFV C18orf8-3xHA pGK Puro | human C18orf8 cloned from HeLa cDNA, C-terminal 3xHA tag | This manuscript |
| pHRSIN.pSFFV 3HA-C18orf8 pGK Puro | as above, N-terminal 3xHA tag | This manuscript |
| pHRSIN.pSFFV C18orf8-mScarlet pGK Puro | as above, C-terminal mScarlet tag; mScarlet cloned from pmScarlet-C1 (a gift from Dorus Gadella, Addgene #85042 [2]) | This manuscript |
| pHRSIN.pSFFV mScarlet-C18orf8(354-657) pGK Puro | as above, C18orf8 C-terminal domain with N-terminal mScarlet tag | This manuscript |
| pHRSIN.pSFFV C18orf8(1-362)-mScarlet pGK Puro | as above, C18orf8 N-terminal domain with C-terminal mScarlet tag | This manuscript |
| pHRSIN.pSFFV Mon1A-3xMyc pGK Hygro | human Mon1A cloned from IMAGE clone (Dharmacon), C-terminal 3xMyc tag | This manuscript |
| pHRSIN.pSFFV Mon1B-3xMyc pGK Hygro | human Mon1B cloned from IMAGE clone (Dharmacon), C-terminal 3xMyc tag | This manuscript |
| pHRSIN.pSFFV Ccz1-3xMyc pGK Hygro | human Ccz1 cloned from IMAGE clone (Dharmacon), C-terminal 3xMyc tag | This manuscript |
| pHRSIN.pSFFV 2xHA-Rab7 pGK Puro | wild-type human Rab7A, cloned from pcDNA3 EGFP-Rab7A (a gift from Qing Zhong, Addgene #28047 [3]), N-terminal 2xHA-tag | This manuscript |
| pHRSIN.pSFFV 2xHA-Rab7-T22N pGK Puro | human Rab7A dominant-negative T22N mutant, cloned from pcDNA3 EGFP-Rab7A-T22N (a gift from Qing Zhong, Addgene #28048 [3]), N-terminal 2xHA-tag | This manuscript |
| pHRSIN.pSFFV 2xHA-Rab7-Q67L pGK Puro | human Rab7A constitutively-active Q67L mutant, cloned from pcDNA3 EGFP-Rab7A-Q67L (a gift from Qing Zhong, Addgene #28049 [3]), N-terminal 2xHA-tag | This manuscript |
| pHRSIN.pCMV 2xHA-Rab7 pGK Puro | as above, wild-type Rab7 with N-terminal 2xHA tag under CMV promoter | This manuscript |
| pHRSIN.pCMV 2xHA-Rab7-T22N pGK Puro | as above, Rab7A-T22N mutant with N-terminal 2xHA tag under CMV promoter | This manuscript |
| pHRSIN.pCMV 2xHA-Rab7-Q67L pGK Puro | as above, Rab7A-Q67L mutant with N-terminal 2xHA tag under CMV promoter | This manuscript |
| pHRSIN.pSFFV EGFP-Rab7 pGK Puro | as above, wild-type Rab7A with N-terminal EGFP-2xHA tag | This manuscript |
| pHRSIN.pSFFV EGFP-Rab7-T22N pGK Puro | as above, Rab7A-T22N mutant with N-terminal EGFP-2xHA tag | This manuscript |
| pHRSIN.pSFFV EGFP-Rab7-Q67L pGK Puro | as above, Rab7A-Q67L mutant with N-terminal EGFP-2xHA tag | This manuscript |
| pHRSIN.pCMV 2xHA-Rab5-Q79L pGK Puro | human Rab5 constitutively-active Q79L mutant, N-terminal 2xHA tag, CMV promoter; Rab5 cloned from mTagBFP2-Rab5A (a gift from Michael Davidson, Addgene #55322 [4]) | This manuscript |
| pHRSIN.pSFFV EGFP-Rab5-S34N pGK Puro | as above, human Rab5A dominant-negative S34N mutant, N-terminal EGFP-2xHA tag | This manuscript |

| | | |
|---|---|---|
| pHRSIN.pCMV 2xHA-Rab9-Q66L pGK Puro | human Rab9A constitutively active Q66L mutant, N-terminal 2xHA tag, CMV promoter; Rab9A cloned from HEK293T cDNA | This manuscript |
| pHRSIN.pSFFV EGFP-Rab9-S21N pGK Puro | as above, Rab9A dominant-negative S21N mutant, N-terminal EGFP-2xHA tag | This manuscript |
| pHRSIN.pCMV 2xHA-Rab11-Q70L pGK Puro | human Rab11A constitutively active Q70L mutant, N-terminal 2xHA tag, CMV promoter; Rab11A cloned from pEGFP Rab11A (a kind gift from Folma Buss, University of Cambridge, UK) | This manuscript |
| pHRSIN.pSFFV EGFP-Rab11-S25N pGK Puro | as above, Rab11A dominant-negative S25N mutant, N-terminal EGFP-2xHA tag | This manuscript |
| pHRSIN.pCMV Arl8B-Q75L-3xHA pGK Puro | human Arl8B constitutively active Q75L mutant, C-terminal 3xHA tag, CMV promoter; Arl8B cloned from pcDNA3 2xMyc-C1 Arl8B [5] | This manuscript |
| pHRSIN.pSFFV Arl8B-T34N-EGFP pGK Puro | as above, Arl8B dominant-negative T34N mutant, C-terminal EGFP tag | This manuscript |
| pcDNA3 HA-RILP | RILP, N-terminal HA-tag for transient expression | [6] |
| pcDNA3 HA-ORP1L | ORP1L, N-terminal HA-tag for transient expression | [7] |
| pHRSIN.pSFFV 3xFLAG-RILP pGK Puro | RILP, N-terminal 3xFLAG tag, lentiviral expression | This manuscript |
| pHRSIN.pSFFV NPC1-CFP pGK Hygro | mouse NPC1, C-terminal CFP tag, cloned from pECFP-N3 NPC1 (a kind gift from Matthew Scott, Addgene #53522 [8]) | This manuscript |
| pHRSIN.pSFFV NPC1-3xHA pGK Hygro | as above, mouse NPC1, C-terminal 3xHA tag | This manuscript |
| pHRSIN.pSFFV NPC1-P692S-3xHA pGK Hygro | mouse NPC1, inactive P692S mutant, C-terminal 3xHA tag; NPC1-P692S cloned from pECFP-N3-NPC1-P692S (a kind gift from Matthew Scott, Addgene #58457 [9]) | This manuscript |
| pHRSIN.pSFFV NPC2-mCherry pGK Puro | human NPC2, C-terminal mCherry tag, cloned from a gBlock | This manuscript |
| pHRSIN.pSFFV FLAG-NLS-CAS9-NLS pSV40 Blast | pSp. CAS9, FLAG-tagged | [10] |
| pHRSIN.pSFFV Cre IRES mCherry pGK Hygro | Bacteriophage P1 Cre recombinase, with IRES mCherry and hygromycin resistance | This manuscript |
| pDonor HMGCS1-Clover pUb Puro | HMGCS1-Clover donor vector for CRISPR knock-in, puromycin resistance; pDonor pUb Puro was a kind gift from Ron Kopito and Matthew Porteus (Stanford University, USA). HMGCS1 5' and 3' donor arms cloned from HeLa genomic DNA using primers below; Clover cloned from pcDNA3 Clover-mRub2 (a gift from Kurt Beam, Addgene #49089 [11]). | This manuscript |
| pDonor HMGCS1-Clover pUb Hygro | as above, HMGCS1-Clover donor vector for CRISPR knock-in, hygromycin resistance | This manuscript |
| pDonor HMGCS1-Clover pSV40 Blast | as above, HMGCS1-Clover donor vector for CRISPR knock-in, blasticidin resistance | This manuscript |
| pDonor C18orf8-3myc pUb Hygro | C18orf8-3myc donor vector for CRISPR knock-in; C18orf8 5' and 3' donor arms cloned from gBlocks (see below) | This manuscript |
| pKLV.U6 pGK Puro-2A-BFP | sgRNA vector, a kind gift from Kosuke Yusa (Wellcome Trust Sanger Institute, UK) | [12] |
| pSpCAS9(BB)-2A-Puro (pX458) | sgRNA vector with pSp. CAS9 (a gift from Feng Zhang, Addgene #48139 [13]) | [13] |
| pSIREN.puro | shRNA vector, a kind gift from Greg Towers (UCL, UK), puromycin resistance | - |
| pSIREN.hygro | as above, shRNA vector, hygromycin resistance | This manuscript |

## shRNA

| shRNA | Sequence | Vector | Ref. |
|---|---|---|---|
| TBC1D5 sh1 | 5'-GAAGCCATATCGCAGAGCTA-3' | pSIREN.puro | [14] |
| TBC1D15 sh1 | 5'-AATGGGACATGGTTAATACAGTT-3' | pSIREN.hygro | [5] |
| non-targeting sh1 | 5'-GGGTATCGACGATTACAAA-3' | pSIREN.puro | - |

## sgRNA

| sgRNA | Sequence | Vector | Ref. |
|---|---|---|---|
| SREBF1 sg1 | 5'-GGTCACAGTGGTCGTTACA-3' | pKLV.U6 pGK Puro-2A-BFP | - |
| SREBF1 sg1 | 5'-GGTCACAGTGGTCGTTACAG-3' | pKLV.U6 pGK Puro-2A-BFP | - |
| SREBF2 sg1 | 5'-AGTGCAACGGTCATTCACCC-3' | pKLV.U6 pGK Puro-2A-BFP | - |
| SREBF2 sg2 | 5'-CTCACCGTCGATGTCTCCCA-3' | pKLV.U6 pGK Puro-2A-BFP | - |
| SREBF2 sg3 | 5'-AGCCGGGCGATGGACGACAG-3' | pKLV.U6 pGK Puro-2A-BFP | - |
| LDLR sg1 | 5'-CGGCGAATGCATCACCC-3' | pKLV.U6 pGK Puro-2A-BFP | - |
| NPC1 sg1 | 5'-CGTCAGCGTCCTTCCCACAC-3' | pKLV.U6 pGK Puro-2A-BFP | - |
| ANKFY1 sg1 | 5'-TATTCGCTTCTACCAGA-3' | pKLV.U6 pGK Puro-2A-BFP | - |
| ANKFY1 sg2 | 5'-TAGCGGGTACTCTGTCT-3' | pKLV.U6 pGK Puro-2A-BFP | - |
| ANKFY1 sg3 | 5'-ACGCAGCCAAACGCCTGTAC-3' | pKLV.U6 pGK Puro-2A-BFP | - |
| ANKFY1 sg4 | 5'-GTACAGGCGTTTGGCTGCGT-3' | pKLV.U6 pGK Puro-2A-BFP | - |
| VPS16 sg1 | 5'-GCAGTGGAAGAGTGGACCCG-3' | pKLV.U6 pGK Puro-2A-BFP | - |
| ZFYVE20 sg1 | 5'-CTCAGCTCTGAATAATCGGG-3' | pKLV.U6 pGK Puro-2A-BFP | - |
| INSIG1 sg1 | 5'-CGTAGCTAGAAAAGCTA-3' | pSpCAS9(BB)-2A-Puro (pX458) | [15] |
| INSIG1 sg2 | 5'-CTGCTGTCCCGCAGCAGGG-3' | pSpCAS9(BB)-2A-Puro (pX458) | [15] |
| INSIG1 sg3 | 5'-CAGCCCCTACCCCAACACC-3' | pSpCAS9(BB)-2A-Puro (pX458) | [15] |
| INSIG1 sg4 | 5'-TCAACCTGCTGCAGATCCAG-3' | pSpCAS9(BB)-2A-Puro (pX458) | [15] |
| C18orf8 sg1 | 5'-GCGGCCGGTGCAGTTCGAGA-3' | pKLV.U6 pGK Puro-2A-BFP | - |
| C18orf8 sg2 | 5'-TTCCGAAAGAGACATCGCAA-3' | pKLV.U6 pGK Puro-2A-BFP | - |
| Mon1A sg1 | 5'-GTTGGGGCCCGTGTAGTAAA-3' | pKLV.U6 pGK Puro-2A-BFP | - |
| Mon1B sg1 | 5'-GCAGGTATAGAGCTCGAATT-3' | pKLV.U6 pGK Puro-2A-BFP | - |
| Ccz1 sg1 | 5'-AATGAGAAGATTAGAAATGT-3' | pKLV.U6 pGK Puro-2A-BFP | - |
| Rab7A sg1 | 5'-AGGCGTTCCAGACGATTGCA-3' | pKLV.U6 pGK Puro-2A-BFP | - |
| β2m sg1 | 5'-GGCCGAGATGTCTCGCTCCG-3' | pKLV.U6 pGK Puro-2A-BFP | [1] |
| HMGCS1 ki sg1 | 5'-TCTGTGAGGTGCAAGACTTC-3' | pSpCAS9(BB)-2A-Puro (pX458) | - |
| C18orf8 ki sg1 | 5'-GAGACAGACAGTGCATTGAT-3' | pSpCAS9(BB)-2A-Puro (pX458) | - |

## Primers for cloning

| Primer | Sequence | Usage |
|---|---|---|
| C18orf8 FW | 5'- GAGTCGCCCGGGGGGGATCCATGGGCGAGGAGGA CTACTA-3' | Cloning full-length C18orf8 |
| C18orf8 RV | 5'- GACTCTAGAGTCGCGGCCGCTCAGAATGTTGTAGG CCTCATT -3' | cloning full-length C18orf8 |
| C18orf8-3xHA RV | 5'- TAATCTGGAACATCATATGGATACTCGAGGAATGT TGTAGGCCTCATT -3' | Cloning C18orf8-3xHA |
| 3xHA-C18orf8 FW | 5'-TGATGTTCCAGATTATGCTGGATCCATG GGCGAGGAGGACTACTA-3' | cloning 3xHA-C18orf8 |
| C18orf8-354 FW | 5'-GAGTCGCCCGGGGGGGATCCATGGGTTACCTCTG GAACCTCCA -3' | cloning C18orf8 (354-657) truncation mutant |
| C18orf8-362-HA RV | 5'-TAATCTGGAACATCATATGGATACTCGAGTTTCACT TGGAGGTTCCAGAG -3' | cloning C18orf8 (1-362) truncation mutant |
| Mon1A FW | 5'-GAGTCGCCCGGGGGGGATCCATGCACCCCGGGGG CGGG-3' | Cloning Mon1A-3xMyc |
| Mon1A-Myc RV | 5'-GAGATGAGTTTTTGTTCCTCGAGATAGGTGAGGGG CGTGAGA-3' | Cloning Mon1A-3xMyc |
| Mon1B FW | 5'-GAGTCGCCCGGGGGGGATCCATGGAGGTCGGAG GAGACA-3' | Cloning Mon1B-3xMyc |
| Mon1B-Myc RV | 5'-GAGATGAGTTTTTGTTCCTCGAGGAGTCCAGTGAA CAAGCCAT-3' | Cloning Mon1B-3xMyc |

## Primers for cloning (continued)

| | | |
|---|---|---|
| Ccz1 FW | 5'-GAGTCGCCCGGGGGGGATCCATGGCTGCAGCGGC GGCC-3' | Cloning Ccz1-3xMyc |
| Ccz1-Myc RV | 5'-GAGATGAGTTTTTGTTCCTCGAGATCCAAGAAGAA GATGTTGTTGA-3' | Cloning Ccz1-3xMyc |
| EGFP FW | 5'-CGCCAGTCCTCCGACAGACTGAGTCGCCCGGGCGC CATGGTGAGCAAGGGCGAGGA -3' | Cloning EGFP-Rab7 constructs |
| Rab7 RV | 5'-AGGTCGACTCTAGAGTCGCGGCCGCTTAGCAACTG CAGCTTTCTGC-3' | Cloning EGFP-Rab7 constructs |
| HA-Rab5 FW | 5'-ACGATGTTCCGGATTACGCTGGTACCGCTAGTCGA GGCGCAACAAG-3' | Cloning 2xHA-Rab5 |
| Rab5 RV | 5'-GACTCTAGAGTCGCGGCCGCTTAGTTACTACAACA CTGATTCCT-3' | Cloning 2xHA-Rab5 |
| Rab5-S34N FW | 5'-GGAGAGTCTGCTGTTGGCAAGAATAGCCTAGTGCT TCGTTTTGTG-3' | Cloning Rab5 S34N mutant |
| Rab5-S34N RV | 5'-CACAAAACGAAGCACTAGGCTATTCTTGCCAACAG CAGACTCTCC-3' | Cloning Rab5 S34N mutant |
| Rab5-Q79L FW | 5'-GAAATATGGGATACAGCTGGTCTAGAACGATACCA TAGCTTAGCA-3' | Cloning Rab5 Q79L mutant |
| Rab5-Q79L RV | 5'-TGCTAAGCTATGGTATCGTTCTTGACCAGCTGTATC CCATATTTC-3' | Cloning Rab5 Q79L mutant |
| HA-Rab9 FW | 5'- CCATACGATGTTCCGGATTACGCTGGTACCGCAGG AAAATCATCACTTTTTAAAG -3' | Cloning 2xHA-Rab9 |
| Rab9 RV | 5'- CCTGCAGGTCGACTCTAGAGTCGCGGCCGCTCAAC AGCAAGATGAGCTAGG -3' | Cloning 2xHA-Rab9 |
| Rab9-S21N FW | 5'- GGTGGAGTTGGGAAGAATTCACTTATGAACAGATA TGTAACTAAT -3' | Cloning Rab9 S21N mutant |
| Rab9-S21N RV | 5'- CTGTTCATAAGTGAATTCTTCCCAACTCCACC ATCTC -3' | Cloning Rab9 S21N mutant |
| Rab9-Q66L FW | 5'-TGGGACACGGCAGGTCTGGAGCGATTCCGAAGCC TGAG -3' | Cloning Rab9 Q66L mutant |
| Rab9-Q66L RV | 5'-CTTCGGAATCGCTCCTGACCTGCCGTGTCCCA AATCT -3' | Cloning Rab9 Q66L mutant |
| HA-Rab11 FW | 5'-CCATACGATGTTCCGGATTACGCTGGTACCGGCAC CCGCGACGACGAGT-3' | Cloning 2xHA-Rab11 |
| Rab11 RV | 5'-CCTGCAGGTCGACTCTAGAGTCGCGGCCGCTTAGA TGTTCTGACAGCACTGC -3' | Cloning 2xHA-Rab11 |
| Rab11-S25N FW | 5'-CTGGTGTTGGAAAGAATAATCTCCTGTCTCGATT TACTC -3' | Cloning Rab11 S25N mutant |
| Rab11-S25N RV | 5'-CGAGACAGGAGATTATTCTTTCCAACACCAG AATCTC-3' | Cloning Rab11 S25N mutant |
| Rab11-Q70L FW | 5'-GGGACACAGCAGGGCTGGAGCGATATCGAGCT ATAACAT-3' | Cloning Rab11 Q70L mutant |
| Rab11-Q70L RV | 5'-AGCTCGATATCGCTCCAGCCCTGCTGTGTCCCA TATCT -3' | Cloning Rab11 Q70L mutant |
| Arl8B FW | 5'-CCGACAGACTGAGTCGCCCGGGGGGGATCCATGC TGGCGCTCATCTCCCGCCTGC-3' | Cloning Arl8B-3xHA |
| Arl8B-HA RV | 5'-CAGAGAAGCATAATCTGGAACATCATATGGATACT CGAGGCTTCTTCTAGATTTTGAGTGTTG -3' | Cloning Arl8B-3xHA |
| Arl8B-T34N FW | 5'-AGTACTCCGGCAAGAATACCTTCGTCAATGTC ATCGC -3' | Cloning Arl8B T34N mutant |
| Arl8B-T34N RV | 5'-GACATTGACGAAGGTATTCTTGCCGGAGTACT GCAGC-3' | Cloning Arl8B T34N mutant |
| Arl8B-Q75L FW | 5'-GACATAGGCGGACTGCCCCGGTTCCGGAGCATGT-3' | Cloning Arl8B Q75L mutant |
| Arl8B-Q75L RV | 5'-CTCCGGAACCGGGGCAGTCCGCCTATGTCCCA GATCT-3' | Cloning Arl8B Q75L mutant |
| FLAG-RILP FW | 5'-TGATGTTCCAGATTATGCTGGATCCGAGCCCAGGA GGGCGGCGC-3' | Cloning 3xFLAG-RILP |

## Primers for cloning (continued)

| | | |
|---|---|---|
| RILP RV | 5'-GACTCTAGAGTCGCGGCCGCTCAGGCCTCTGGGGC GGCT-3' | Cloning 3xFLAG-RILP |
| NPC1-KpnI-XhoI linker FW | 5'-CGCGGGCCCGGGATCCATCGCCACCC-3' | Cloning NPC1-3xHA |
| NPC1-KpnI-XhoI linker RV | 5'-TCGAGGGTGGCGATGGATCCCGGGCCCGCGGTAC-3' | Cloning NPC1-3xHA |
| HMGCS1 5'arm FW | 5'-TGCCCTTGGGCTCCCCGGGCGCGACTAG TGAATTCGGTTCATTTGTGGTGTGTGTCT-3' | cloning pDonor HMGCS1-Clover pUb Puro knock-in donor vector |
| HMGCS1 5'arm RV | 5'-TCTTCTGAGATGAGTTTTTGTTCAGAACC CGATCGATGTTCCCCATTACTAATGAC-3' | cloning pDonor HMGCS1-Clover pUb Puro knock-in donor vector |
| HMGCS1 3'arm FW | 5'-CGTATAGCATACATTATACGAAGTTATTT AATTAAGTGGGGTGGGCATGGGGTG-3' | cloning pDonor HMGCS1-Clover pUb Puro knock-in donor vector |
| HMGCS1 3'arm RV | 5'-CCGCATTCTTATAATCAGCATCATGATGT GGTACCTGCAACAACAAACTCCCTCA-3' | cloning pDonor HMGCS1-Clover pUb Puro knock-in donor vector |

## Primers for CRISPR screening

| Primer | Sequence | Usage |
|---|---|---|
| Top1000 sublibr FW | 5'-AGGCACTTGCTCGTACGACG-3' | amplification of targeting sgRNAs for cloning Top 1000 sgRNA library |
| Top1000 sublibr RV | 5'-ATGTGGGCCCGGCACCTTAA-3' | amplification of targeting sgRNAs for cloning Top 1000 sgRNA library |
| Ctrl sg sublibr FW | 5'-GTGTAACCCGTAGGGCACCT-3' | amplification of non-targeting sgRNAs for cloning Top 1000 sgRNA library |
| Ctrl sg sublibr RV | 5'-GTCGAGAGCAGTCCTTCGAC-3' | amplification of non-targeting sgRNAs for cloning Top 1000 sgRNA library |
| Bassik sgRNA PCR1 FW | 5'-AGGCTTGGATTTCTATAACTTCGTATAGC ATACATTATAC-3' | PCR1 for integrated sgRNA in genome-wide CRIPR screen |
| Bassik sgRNA PCR1 RV | 5'-ACATGCATGGCGGTAATACGGTTATC-3' | PCR1 for integrated sgRNA in genome-wide CRIPR screen |
| Bassik sgRNA PCR2 FW | 5'-AATGATACGGCGACCACCGAGATCTACA CGCACAAAAGGAAACTCACCCT-3' | PCR2 for integrated sgRNA in genome-wide CRIPR screen |
| Bassik sgRNA PCR2 RV | 5'-CAAGCAGAAGACGGCATACGAGAT nnnnnnnGTGACTGGAGTTCAGACGTGTGCTCTTCCGAT CCGACTCGGTGCCACTTTTTC-3' | PCR2 for integrated sgRNA in genome-wide CRIPR screen, nnnnnn indicates variable Illumina index sequence |
| Bassik seq | 5'-AGACTATAAGTATCCCTTGGAGAACCACCTTG TTGG-3' | Illumina sequencing of genome-wide CRIPR screen |
| Top1000 Libr PCR1 FW | 5'-GCTTACCGTAACTTGAAAGTATTTCG-3' | PCR1 for integrated sgRNA in targeted top 1000 CRIPR screen |
| Top1000 Libr PCR1 RV | 5'-CGAGACTAGTGAAACGTGCTAC-3' | PCR1 for integrated sgRNA in targeted top 1000 CRIPR screen |
| Top1000 Libr PCR2 FW | 5'-AATGATACGGCGACCACCGAGATCTACA CTCTCTTGTGGAAAGGACGAAACACCG-3' | PCR2 for integrated sgRNA in targeted top 1000 CRIPR screen |
| Top1000 Libr PCR2 RV | 5'-CAAGCAGAAGACGGCATACGAGAT nnnnnnnGTGACTGGAGTTCAGACGTGTGCTCTTCCGAT CT CCATTTGTCACGTCCTGCACG-3' | PCR2 for integrated sgRNA in targeted top 1000 CRIPR screen, nnnnnn indicates variable Illumina index sequence |
| Top1000 seq | 5'- CTTGTGGAAAGGACGAAACACCG-3' | Illumina sequencing of targeted top 1000 CRIPR screen |

## Gene fragments

| gBlock | Sequence | Usage |
|---|---|---|
| NPC2-mCherry | 5’-GAGTCGCCCGGGGGGGATCCATGCGTTTTCTGGCCGCCACGATCCTGCTGCTGGCGCTGGTCGCTGCCAGCCAGGCGGAGCCCCTGCA<br>CTTCAAGGACTGCGGCTCTAAGGTGGGAGTTATAAAGGAGGTGAATGTGAGCCCATGTCCCACCGATCCCTGTCAGCTGCACAAAGGCCAG<br>TCCTACAGTGTCAACATCACCTTTACCAGCGGCACTCAGTCCCAGAACAGCACGGCCTTGGTCCACGGCATCCTGGAAGGGATCCGGGTCCC<br>CTTCCCTATTCCTGAGCCTGACGGTTGTAAGAGTGGAATCAACTGCCCCATCCAGAAAGACAAGGTCTACAGCTACCTGAATAAGCTTCCGGT<br>GAAGAATGAATACCCCTCTATAAAACTGGTGGTGGAATGGAAACTTGAAGATGACAAAAAGAATAATCTCTTCTGCTGGGAGATCCCAGTTC<br>AGATCACAAGCCCGCCTCCCCCTCCTCCACCTCCACCTCCCATCATCAAGGAGTTCATGCGCTTCAAGGTGCACATGGAGGGCTCCGTGAACG<br>GCCACGAGTTCGAGATCGAGGGCGAGGGCGAGGGCCGCCCCTACGAGGGCACCCAGACCGCCAAGCTGAAGGTGACCAAGGGTGGCCCC<br>CTGCCCTTCGCCTGGGACATCCTGTCCCCTCAGTTCATGTACGGCTCCAAGGCCTACGTGAAGCACCCCGCCGACATCCCCGACTACTTGAAG<br>CTGTCCTTCCCCGAGGGCTTCAAGTGGGAGCGCGTGATGAACTTCGAGGACGGCGGCGTGGTGACCGTGACCCAGGACTCCTCCCTGCAGG<br>ACGGCGAGTTCATCTACAAGGTGAAGCTGCGCGGCACCAACTTCCCCTCCGACGGCCCCGTAATGCAGAAGAAGACCATGGGCTGGGAGGC<br>CTCCTCCGAGCGGATGTACCCCGAGGACGGCGCCCTGAAGGGCGAGATCAAGCAGAGGCTGAAGCTGAAGGACGGCGGCCACTACGACGC<br>TGAGGTCAAGACCACCTACAAGGCCAAGAAGCCCGTGCAGCTGCCCGGCGCCTACAACGTCAACATCAAGTTGGACATCACCTCCCACAACG<br>AGGACTACACCATCGTGGAACAGTACGAACGCGCCGAGGGCCGCCACTCCACCGGCGGCATGGACGAGCTGTACAAGTAAGCGGCCGCGA<br>CTCTAGAGTCGACCTGCA-3’ | Cloning NPC2-mCherry construct (design based on [16]) |
| C18orf8 5’arm | 5’-TGCCCTTGGGCTCCCCGGGCGCGACTAGTGAATTCAAGCCAGGAATAAGACGGCAAAT<br>GGTGGCTAAGGGTTTTAAAGGGTCTGGGGCTTATTAAGGTTTCAGTTTTATGAAGTATACATTGGTTGATCATTCTCATTTTTTTTTGAGACA<br>GAGTCTCGCTCCGTTGCCCAGGCTGGAGTGCAGTGGTGTGATCTCGGTTCACTGCAACCTCCGCTGGGTTCAAGCGATTCTTCTGCCTCAGCC<br>TCCTGAGTAGTTGGGACTACAGGTGCGTGCCACCACACCCGGCTGATTTTTGTATTTTTAGTAGAGATGGGGTTTTACTATATTGTCCAGGCT<br>GGTCTCAAACTCCTGACCTCATGATCCGCCTGCCTCAGCCTCCCAAAGTGCTGGGATTACAGGTTTGAGCCACCGCACCTGGCCCTGGTTGAT<br>CATTCTCATCTCCAAATAGTTATACAACCTAAGACATCATCTTTAGGATAGGGAAGGATCAGGGCTGTCTAATGAAACTTCTAGGTCTATTTCT<br>AGCTCAATGTAAGACGGCATTTAGTTACCATAAGGACAGGTTAGATAGAATCTCTTCCATTTAGCTTTTGTATTTGTCTCTCAAAGCAGATAG<br>GGTAACCCCAAAACTTAGGAAAACAATGTATTTTATTAAAGAAAAATAAGTTAAAACCCAGTAGACACACCTACGAGATGCTTTCTTTGTCCC<br>TCATTTCATGCCACATCTAACTGGCAATTAAATCTCTTCCTTTCTAGGGGAACACTGTGAAGAACATGTTGCTTTTTTCAAACAGATTTTTGGA<br>GACCAAGCTCTAATGAGGCCTACAACATTCCGATCGGAACAAAAACTCATCTCAGAAGAGGATCTG-3’ | cloning pDonor C18orf8-3myc pUb Hygro knock-in donor vector |
| C18orf8 3’arm | 5’-CGTATAGCATACATTATACGAAGTTATTTAATTAACCATAGAGCCCACCGCATCCCCAG<br>CATGCCTGCTATTGTCTTCCCAATCCTCCCCCTTGCTGTCCTGCCCCACCCCACCCCCCAGAATAGAATGACACCTACTCAGACAATGCGATGC<br>AATTTCCTCATTTTATTAGGAAAGGACAGTGGGAGTGGCACCTTCCAGGGTCAAGGAAGGCACGGGGGAGGGGCAAACAACAGATGGCTG<br>GCAACTAGAAGGCACAGTTTAATTAAGGCGCGCCTGGCCTTTACAGAGTGTCAGTGAGCGGATCACATTCACAGCCATCTAGTGTCACCTCC<br>TGCTTGCCAAAGAATGAGGTTTCTTTCCTGAAGAGGCTGGGAGAAGTTTAGTGTCCTGTGGTTGCCTCCAGATCTAGTAATACTGCTTCCCAA<br>GTCAACTGTGCATTCCTGAGTTCACAGGCGCTACGTTCAAAGCTGCTGCCAAACAACCGATGGTTGGCACCATCCGGTGTTCAACTTGGCCTT<br>GCCGATGCAGCACCCGTCCAGTGGTAAACCGACCGACCCTTAGACACAGTTCAGTCAGGATGCCCTGCGAGAGGGCTAGAAATTTAGAAGC<br>CGTTCGCGCTCTGTTCCTTTGTATCGCTCTTCAGTGGCACAACTTTTGGCTTTATTTACTGATGGCCCTATGAGAGAGAGAGACTTTTTCTTATT<br>TCTGCAGGAGAAAGGGAGCTAGTTGGCTGGGCATAGTGGCTCACGCCTGTAATCCCACTTTGGAAGACTGAGGCAGGAGAATTGCTTGAGA<br>CCAGCCTGGACAACAGAGTGAGACCACATCTCTCTCCCTCTCTTTTGGAGATGGGCTCTTTGCTTTCTTCCTCAGGCTGCAGTGCAGTGGTAT<br>GAACACGACTCACTGCATCCTAAGCCCACCCTTCTGGGCTCAAGCAAGCTTCCTGCCTCAGCCTAACAAGTAGGTGGGACTGCAGACGCACA<br>CCACTTCACCTGGCTAATTTTATTTTTGTAGAGATGGAGGCCTCACTATGTTGCCTAGGCTGGTCTCAAACGATTCTCCTGTCTCAGCCTTCCA<br>AGGTACCACATCATGATGCTGATTATAAGAATGCGG-3’ | cloning pDonor C18orf8-3myc pUb Hygro knock-in donor vector |

## Antibodies

| Antibody | Supplier | Catalogue No. | Usage (dilution) |
|---|---|---|---|
| mouse anti-HA-tag (16B12) | Biolegend | #901501 | WB (1:10000) |
| rabbit anti-HA-tag (Poly9023) | Biolegend | #923501 | WB (1:10000) |
| rat anti-HA-tag (3F10) | Sigma-Aldrich | #ROAHAHA | WB (1:5000), IF (1:300) |
| rabbit anti-Myc-tag (71D10) | Cell Signalling | #2278 | WB (1:2000) |
| mouse anti-Myc-tag (9B11) | Cell Signalling | #2276 | WB (1:10000) |
| mouse anti-FLAG-tag (M2) | Sigma-Aldrich | #F1804 | WB (1:10000) |
| rabbit anti-GFP-tag | Thermo-Fisher | #A11122 | WB (1:10000), IF (1:500) |
| mouse anti-RFP-tag (6G6) | Chromotec | #6g6-100 | WB (1:2500) |
| rat anti-RFP-tag (5F8) | Chromotec | #5f8-100 | IP (1:200) |
| rabbit anti-Rab5 (C8B1) | Cell Signalling | #3547 | IF (1:200) |
| rabbit anti-Rab7 (D95F2) | Cell Signalling | #9367 | WB (1:5000), IF (1:100) |
| mouse anti-Rab7 (E9O7E) | Cell Signalling | #95746 | IP (1:500) |
| rabbit anti-Rab7 (EPR7589) AlexaFluor647 conjugate | Abcam | #ab198337 | IF (1:100) |
| mouse anti-C18orf8 (OTI4E4) | Novus | #NBP2-01949 | WB (1:2000), IP (1:500) |
| rabbit anti-C18orf8 | Proteintech | #20111-1-AP | WB (1:1000) |
| rabbit anti-Mon1B | Novus | #NBP1-92131 | WB, IP (1:2000) |
| rabbit anti-Mon1B | Proteintech | #17638-1-AP | WB (1:1000) |
| mouse anti-Ccz1 (B-7) | Santa Cruz | #sc-514290 | WB, IP (1:500) |
| mouse anti-EEA1 (14/EEA1) | BD Biosciences | #610456 | IF (1:200) |
| rabbit anti-NPC1 | Novus | NB400-148 | WB (1:2000), IP (1:1000) |
| mouse anti-LAMP1 (H4A3) | Biolegend | #328601 | IF (1:200) |
| mouse anti-LAMP1 (H4A3) BV421 conjugate | BD Biosciences | #562623 | IF (1:200) |
| mouse anti-LAMP1 (H4A3) AlexaFluor647 conjugate | Biolegend | #121610 | IF (1:200) |
| mouse anti-CD63 (MEM-259) | Thermo-Fisher | #MA1-19281 | IF (1:200) |

## Antibodies (continued)

| | | | |
|---|---|---|---|
| mouse anti-HMGCS1 (A6) | Santa Cruz | #sc-166763 | WB (1:1000), IP (1:200) |
| mouse anti-HMGCR (C1) | Santa Cruz | #sc-271595 | IP (1:200) |
| mouse anti-LDLR (C7) PE-conjugate | BD Biosciences | #565653 | FC (1:100) |
| mouse anti-alpha-tubulin (DM1A) | Thermo-Fisher | #62204 | WB (1:20000) |
| mouse anti-beta-actin (AC-74) | Sigma-Aldrich | #A5316 | WB (1:20000) |
| rabbit anti-BODIPY-FL | Thermo-Fisher | #A5770 | EM (1:200) |
| goat-anti-rabbit IgG (H+L) AlexaFluor546 conjugate | Thermo-Fisher | #A11035 | IF (1:500) |
| goat-anti-rabbit IgG (H+L) AlexaFluor647 conjugate | Thermo-Fisher | #A32733 | IF (1:500) |
| goat-anti-mouse IgG (H+L) AlexaFluor568 conjugate | Thermo-Fisher | #A11031 | IF (1:500) |
| goat-anti-mouse IgG (H+L) AlexaFluor647 conjugate | Thermo-Fisher | #A21236 | IF (1:500) |
| goat-anti-mouse IgG1 AlexaFluor546 conjugate | Thermo-Fisher | #A21123 | IF (1:500) |
| goat-anti-rat IgG (H+L) AlexaFluor546 conjugate | Thermo-Fisher | #A11081 | IF (1:500) |
| donkey anti-mouse IgG (H+L) AlexaFluor647 | Thermo-Fisher | #A31571 | IF (1:500) |
| donkey anti-rat IgG (H+L) CF568 conjugate | Biotium | #20092 | IF (1:200) |
| goat-anti-mouse IgG2a BV421 conjugate | Jackson Immunoresearch | #115-675-206 | IF (1:200) |
| goat-anti-mouse (H+L) HRP-conjugate | Jackson Immunoresearch | #115-035-146 | WB (1:25000) |
| goat-anti-rabbit (H+L) HRP-conjugate | Jackson Immunoresearch | #111-035-144 | WB (1:25000) |
| mouse-anti-rat (H+L) HRP-conjugate | Jackson Immunoresearch | #212-035-168 | WB (1:10000) |
| mouse TrueBlot ULTRA HRP-conjugate | Rockland | #18-8817-33 | WB (1:5000) |
| EZview Red anti-HA Affinity Gel | Sigma-Aldrich | #E6779 | IP |
| EZview Red anti-c-Myc Affinity Gel | Sigma-Aldrich | #E6654 | IP |
| EZview Red anti-FLAG M2 Affinity Gel | Sigma-Aldrich | #F2426) | IP |
| GFP-trap magnetic agarose | Chromotec | #gtma-10 | IP |
| Protein A-Sepharose | Sigma-Aldrich | #P3391 | IP |
| Protein G-Sepharose | Sigma-Aldrich | #P3296 | IP |
| IgG sepharose 6 Fast Flow | GE Healthcare | #GE17-0969-01 | IP |

(WB = Western blotting, IP = Immune precipitation, IF = Immune fluorescence, EM = Electron Microscopy, FC = Flow cytometry)

## References


1. van den Boomen, D.J. *et al.* TMEM129 is a Derlin-1 associated ERAD E3 ligase essential for virus-induced degradation of MHC-I. *Proc Natl Acad Sci U S A* **111**, 11425-11430 (2014).
2. Bindels, D.S. *et al.* mScarlet: a bright monomeric red fluorescent protein for cellular imaging. *Nat Methods* **14**, 53-56 (2017).
3. Sun, Q., Westphal, W., Wong, K.N., Tan, I. & Zhong, Q. Rubicon controls endosome maturation as a Rab7 effector. *Proc Natl Acad Sci U S A* **107**, 19338-19343 (2010).
4. Subach, O.M., Cranfill, P.J., Davidson, M.W. & Verkhusha, V.V. An enhanced monomeric blue fluorescent protein with the high chemical stability of the chromophore. *PLoS One* **6**, e28674 (2011).
5. Jongsma, M.L. *et al.* SKIP-HOPS recruits TBC1D15 for a Rab7-to-Arl8b identity switch to control late endosome transport. *EMBO J* **39**, e102301 (2020).
6. van der Kant, R. *et al.* Late endosomal transport and tethering are coupled processes controlled by RILP and the cholesterol sensor ORP1L. *J Cell Sci* **126**, 3462-3474 (2013).
7. Wijdeven, R.H. *et al.* Cholesterol and ORP1L-mediated ER contact sites control autophagosome transport and fusion with the endocytic pathway. *Nat Commun* **7**, 11808 (2016).
8. Lopez, M.E., Klein, A.D., Dimbil, U.J. & Scott, M.P. Anatomically defined neuron-based rescue of neurodegenerative Niemann-Pick type C disorder. *J Neurosci* **31**, 4367-4378 (2011).
9. Ko, D.C., Gordon, M.D., Jin, J.Y. & Scott, M.P. Dynamic movements of organelles containing Niemann-Pick C1 protein: NPC1 involvement in late endocytic events. *Mol Biol Cell* **12**, 601-614 (2001).
10. Timms, R.T. *et al.* Genetic dissection of mammalian ERAD through comparative haploid and CRISPR forward genetic screens. *Nat Commun* **7**, 11786 (2016).
11. Lam, A.J. *et al.* Improving FRET dynamic range with bright green and red fluorescent proteins. *Nat Methods* **9**, 1005-1012 (2012).
12. Koike-Yusa, H., Li, Y., Tan, E.P., Velasco-Herrera Mdel, C. & Yusa, K. Genome-wide recessive genetic screening in mammalian cells with a lentiviral CRISPR-guide RNA library. *Nat Biotechnol* **32**, 267-273 (2014).
13. Ran, F.A. *et al.* Genome engineering using the CRISPR-Cas9 system. *Nat Protoc* **8**, 2281-2308 (2013).
14. Jia, D. *et al.* Structural and mechanistic insights into regulation of the retromer coat by TBC1d5. *Nat Commun* **7**, 13305 (2016).
15. Menzies, S.A. *et al.* The sterol-responsive RNF145 E3 ubiquitin ligase mediates the degradation of HMG-CoA reductase together with gp78 and Hrd1. *Elife* **7** (2018).
16. Huang, L., Pike, D., Sleat, D.E., Nanda, V. & Lobel, P. Potential pitfalls and solutions for use of fluorescent fusion proteins to study the lysosome. *PLoS One* **9**, e88893 (2014).
